# An unusual presentation of cutaneous histoplasmosis as a recurrent solitary and spontaneously healing lesion in an immunocompetent patient

**DOI:** 10.1099/acmi.0.000156

**Published:** 2020-07-21

**Authors:** Jessica L. Muldoon, Timothy R. Wysozan, Yulianna Toubin, Ryan F. Relich, Thomas E. Davis, Chen Zhang, Ahmed K. Alomari

**Affiliations:** ^1^​ Indiana University School of Medicine, Indianapolis, IN, USA; ^2^​ Pathology, Indiana University School of Medicine, Indianapolis, IN, USA; ^3^​ Dermatology, Indiana University Health Methodist Hospital, Indianapolis, IN, USA; ^4^​ Dermatopathology, Indiana University School of Medicine, Indianapolis, IN, USA

**Keywords:** dermatopathology, fungal infections, *Histoplasma*

## Abstract

Infection with *Histoplasma capsulatum* typically manifests as a self-limiting pulmonary disease in immunocompetent patients. Systemic symptoms such as cutaneous lesions are associated with immunodeficient states. Our patient was an immunocompetent 68-year-old male who presented with a plaque on his left infraorbital area that was concerning for malignancy. Histological examination of the lesion revealed granulomatous inflammation and small yeast forms suggestive of *H. capsulatum*. The lesion resolved spontaneously and recurred 1 year later. On recurrence, histological examination again revealed yeast forms consistent with *H. capsulatum*. Serum and urine testing for *H. capsulatum* antigen were negative. Next-generation sequencing detected *H. capsulatum*, which supported the diagnosis of a cutaneous infection. The patient was prescribed and started treatment with itraconazole for 1 year after recurrence of the lesion, and he has not reported further disease recurrence to date. This case is unique because of the presentation of a primary cutaneous recurrent *H. capsulatum* lesion, and it demonstrated the utility of laboratory testing in its diagnosis.

## Introduction

*Histoplasma capsulatum*, a member of the family *Ajellomycetaceae*, is a pathogenic dimorphic fungus that is endemic to the Ohio and Mississippi River Valleys of North America. Typically, *H. capsulatum* causes a self-limiting pulmonary disease in immunocompetent individuals; however, it can cause often-severe systemic disease in the immunosuppressed. People are most commonly infected subsequent to inhalation of microconidia that are produced by the mould-form of the organism, which grows in nature. In the lungs, conidia are engulfed by alveolar macrophages and organisms convert to yeast forms that replicate and persist intracellularly [1]. Primary infection precipitates an influenza-like illness that is marked by cough, fatigue, headache and fever. Large inocula or prior pulmonary disease can predispose the patient to more severe pulmonary disease [2]. Systemic disease has various presentations, including, but not limited to, weight loss, bone marrow failure, liver and spleen involvement, and cutaneous manifestations [2]. Focal infections are most commonly treated with an azole antimicrobial such as itraconazole, while systemic infections are treated with amphotericin B.

Primary cutaneous lesions that present without systemic disease are rare and are often the result of direct inoculation of conidia. Cutaneous lesions, whether primary or secondary to systemic involvement, are variable in presentation, and include papules, plaques, nodules, ulcers and dermatitis [3]. Histopathological characteristics within the dermis include intracellular and/or extracellular yeast forms that are surrounded by colourless halos when observed in haematoxylin and eosin (H&E)-stained sections. *H. capsulatum* is also associated with granulomatous inflammation [4]. Here, we present a case of histoplasmosis presenting and recurring as a solitary plaque, and we discuss these two potential routes of pathogenesis in light of mixed radiological and clinical laboratory findings.

## Case report

**Fig. 1.**
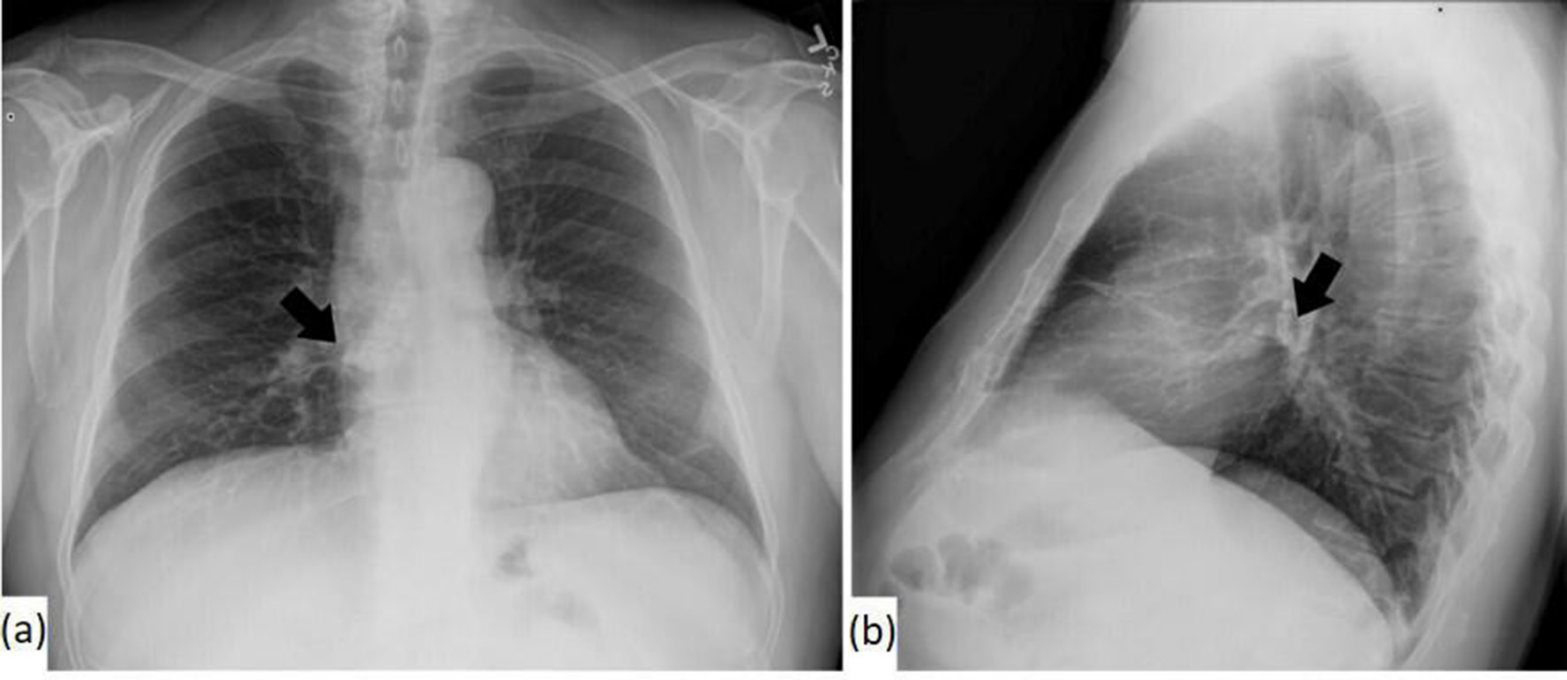
Chest x-ray: (a) posteroanterior and (b) lateral. Small calcified nodes were present in the inferior right hilum and subcarinal chain (indicated by the arrow). No suspicious pulmonary nodules nor infiltrates were identified.

**Fig. 2.**
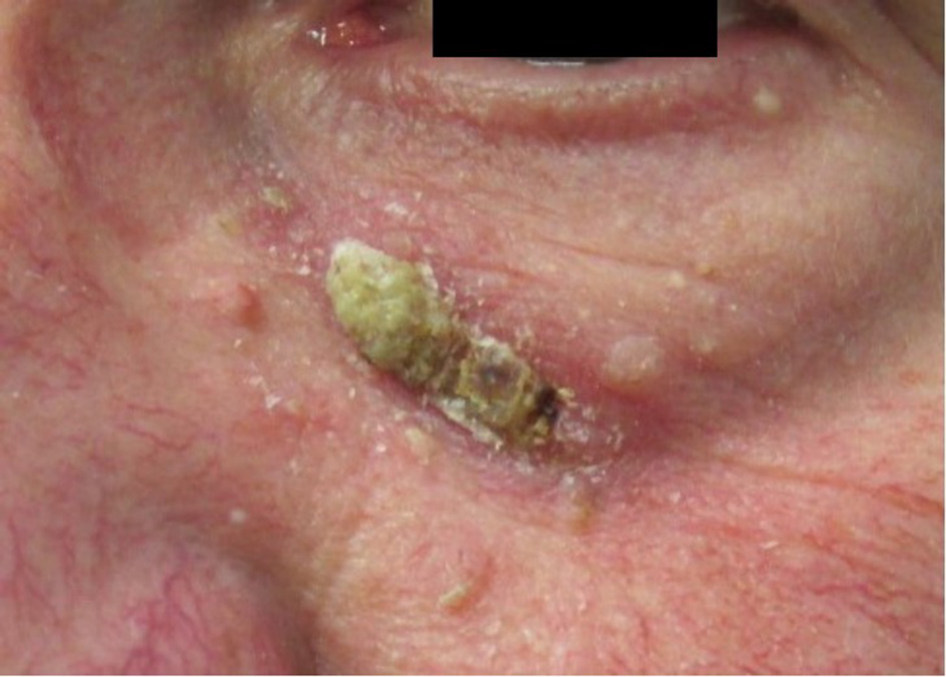
The 2.0×1.0 cm mildly erythematous thin plaque with a yellow crust.

**Fig. 3.**
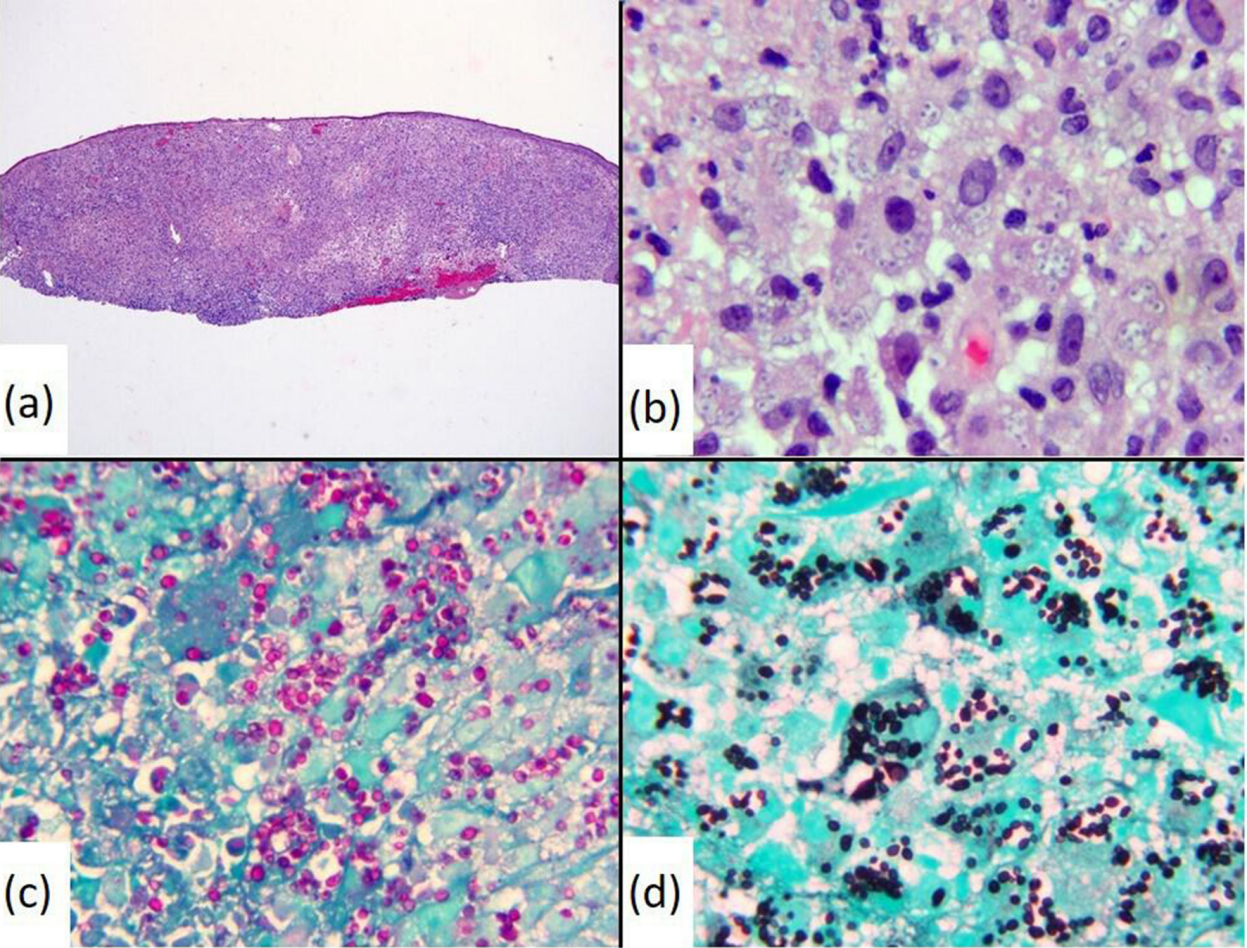
H&E stain of the second tissue biopsy revealed granulomatous inflammation and numerous yeast cells within macrophages, ×400 (a) and ×1000 (b) original magnifications. PAS (c) and GMS (d) stains of the tissue highlighted the presence of the yeast, ×1000 original magnifications.

A 68-year-old male presented with a 2.0×0.7 cm crusted keratotic plaque on his left infraorbital area that was concerning for nonmelanoma skin cancer. An H&E-stained section of a shave-biopsy revealed granulomatous inflammation with pseudoepitheliomatous hyperplasia and a few yeast with narrow-based budding suggestive of *H. capsulatum*. Urine testing for *H. capsulatum* antigen was negative. This lesion resolved within 2 weeks without medical intervention. Chest radiographs revealed small minimally calcified lymph nodes in the inferior right hilum and subcarinal chain (Fig. 1). One year later, the patient presented with recurrence of the lesion as a 2.0×1.0 cm thin, irritated, erythematous and crusted plaque (Fig. 2). No preceding trauma nor recent change in health status nor medications were reported. A full-skin examination demonstrated no similar lesions. Repeat biopsy was performed and showed findings similar to those previously described; however, numerous yeast forms were noted in this specimen (Fig. 3a, b). The organisms were highlighted by periodic acid–Schiff (PAS) and Grocott–Gomori methenamine silver (GMS) stains (Fig. 3c, d). Negative Fontana–Masson and mucicarmine stains excluded the presence of a *Cryptococcus* sp. Overall, the morphological appearance and the results of the special histological stains were consistent with those expected for *H. capsulatum* infection. Extensive microbiological testing, including *Histoplasma* H and M bands via immunodiffusion, urine *Histoplasma* antigen, *Histoplasma* mycelia complement fixation, *Histoplasma* yeast complement fixation, serum *Blastomyces* antigen and antibody, serum *Aspergillus* antibody, serum *Coccidian* antibody, and serum HIV antibodies and antigens were unrevealing. Cultivation of an aetiological agent was hampered by mishandling of the specimen prior to its receipt by the laboratory. A respiratory culture was not performed given significant delays in scheduling. Next-generation nucleic acid sequencing (NGS) of a 230 bp region of the internal transcribed spacer (ITS) that was amplified from the tissue block confirmed the presence of *H. capsulatum*. NGS was performed using an iSeq100 system (Illumina). Sequence data were analysed using an online database (ITS metagenomics app v. 1.1.0 at BaseSpace Sequence Hub). The patient was prescribed and started treatment with 200 mg itraconazole daily for a duration of 1 year and, to date, he has not reported recrudescence of the infection 8 months following his second biopsy.

## Discussion

*H. capsulatum* is endemic to the Ohio and Mississippi River Valleys of North America. Human infection commonly presents as a mild, self-limiting influenza-like disease in immunocompetent patients [1, 5]. Evidence of prior exposure can be determined by delayed-type hypersensitivity or pulmonary calcifications [1]. Immunosuppressed patients are more likely to develop systematic symptoms that can involve several organ systems and can sometimes be fatal [1].

While cutaneous lesions are often seen with disseminated histoplasmosis, they can also present as localized, primary lesions after direct inoculation [6, 7]. A wide variety of lesions that are associated with primary or secondary histoplasmosis have been reported, including nodules, plaques, papules, pustules, dermatitis and cellulitis [3]. Primary cutaneous cases are also often associated with immunosuppression, but have been occasionally reported in immunocompetent patients (Table 1) [7–9].

**Table 1.** Summary of cases of primary (or likely primary) cutaneous histoplasmosis previously reported in the literature

Reference	Age (years)/gender	Clinical presentation	Special stain	Fungal culture result
This case report	68/M	Crusty thin plaque on the left infraorbital area	PAS and GMS stain positive; mucicarmine negative; Fontana–Masson negative	Not performed due to specimen mishandling
Pal and Adhikary [7]	28/F	Multiple nodulo-ulcerative lesions on neck and chest	PAS positive	No growth, patient was on antifungals for 1 week prior
Radhakrishnan *et al*. [8]	46/M	Pigmented ulcerative lesion along the right upper eyelid	PAS positive	No growth
Singhi *et al.* [9]	60/F	Multiple erythematous nodules and plaques over the neck, chest and abdomen	PAS positive	Cottony white mycelium consistent with *H. capsulatum*

F, Female; M, male.

*H. capsulatum* antigen is more likely to be detectable in the urine of patients with disseminated (92–95 %) or acute pulmonary diseases (75%) [2], which were absent in our patient, providing a possible explanation for the negative results of urine (and serum)-based testing. In our patient, chest radiographs showed small minimally calcified lymph nodes. These could be incidental, in which case the cutaneous disease represents a true primary infection as a result of direct inoculation. Alternatively, the presence of the calcified nodes could have signified past *H. capsulatum* pulmonary infection with limited cutaneous reactivation due to unknown factors. Importantly, the patient’s negative systemic work-up argues against an ongoing systemic infection.

While there have been reports of primary cutaneous *H. capsulatum* infections in immunocompetent patients, this case is unique in its clinical presentation of spontaneous resolution and seemingly unprovoked recurrence. This case also highlights the practical use of various radiological, histological and clinical laboratory tests in establishing the diagnosis where the clinical presentation is atypical.
